# Supplementary material for: Whole-body protein kinetics in critically ill patients during 50 or 100% energy provision by enteral nutrition: A randomized cross-over study
Source: PLoS One. 2020 Oct 5;15(10):e0240045. doi: 10.1371/journal.pone.0240045 (PMC7535026; doi:10.1371/journal.pone.0240045)
Supplement: S1 File — (PDF) [file pone.0240045.s002.pdf]

Regionala Etikprövningsnämnden i Stockholm

2016/76-31

Allmänt

Diarienr.:	76
Gruppnr.:	31
In/Ut:	2016-01-19
Ärendetyp:	Ink
Avgift:	5000 2016-01-18
Avdelning:	Medicin
Ansökan komplett:	2016-01-29
Prövn.tillfälle 1:	2016-02-17
Föredragande 1:	Gabriela Spulber
Komplettering 1 ink.:	
Prövn.tillfälle 2:	
Komplettering 2 ink.:	
Prövn.tillfälle 3:	
Föredragande 2:	
Beslut:	Godkännes med villkor
**Exp.datum:**	**2016-02-23**
Överkl.:	☐

Projekttitel/Ärendebeskrivning

Helkropps proteinomsättning hos kritiskt sjuka patienter med hypokalorisk och normokalorisk enteral nutrition

Samband Dnr

Forskningshuvudman/Avs/Mott

Avs/mottagare:	Stockholms läns landsting
Adress:	Karolinska universitetssjukhuset
Postadress:	141 86 Stockholm

Företrädare forskningshuvudmannen

Namn:	Patrik Rossi
Adress:	Anestesikliniken
Postadress:	141 86 Stockholm

Forskare

Namn:	Jan Wernerman
Adress:	Anestesikliniken
Postadress:	141 86 Stockholm
Telefon:	08-58586395, 070-7461000
E-post:	jan.wernerman@karolinska.se

Ordförande
Annika Marcus

Ledamöter med vetenskaplig kompetens
Sigurd Vitols, (klinisk farmakologi), **vetenskaplig sekreterare**
Britt Gustafsson (barnmedicin)
Lene Lindberg (psykologi, folkhälsovetenskap)
Tommy Linné (barnmedicin)
Erik Näslund (kirurgi)
Jonas Persson (psykologi)
Joy Roy (kärlkirurgi)
Agneta Rydberg (klinisk neurovetenskap, oftalmologi)
Gabriela Spulber (geriatrik) , deltar inte i ärendena 2016/95, 2016/96, 2016/97, 2016/104, 2016/106, 2016/107, 2016/115, 2016/124, 2016/135, 2016/137, 2016/145, 2016/149 och 2016/150
Lars Ährlund-Richter (experimentell cancerforskning, utvecklingsbiologi), deltar inte i ärende 2016/42

Ledamöter som företräder allmänna intressen
Catarina Agrell
Lisbeth Crabo Ljungman
Sven Kinnander

Administrativ sekreterare
Eja Fridsta

§ **1** Ordföranden förklarar sammanträdet öppnat.

§ **2** Ansökningar om etisk granskning av forskningsprojekt, se **Bilaga.**

§ **3** Ordföranden förklarar sammanträdet avslutat och meddelar att nästa sammanträde i avdelning 4 äger rum onsdagen den 16 mars 2016.

Annika Marcus
Ordförande

Sigurd Vitols, protokollförare
Vetenskaplig sekreterare

Postadress
FE 289
171 77 STOCKHOLM

Org. nummer
202200 1578

Besöksadress
Tomtebodavägen 18 A, Hiss 1, plan 3
171 65 SOLNA

Telefon
08-524 870 00

Fax
08-524 866 99

E-Post
kansli@stockholm.epn.se

Webb
www.epn.se

Diarienummer:
2016/76-31/4
Föredragande:
Gabriela Spulber

Sökande: Stockholms läns landsting
Behörig företrädare: Patrik Rossi
Projekt: Helkropps proteinomsättning hos kritiskt sjuka patienter med hypokalorisk och normokalorisk enteral nutrition
Forskare som genomför projektet: Jan Wernerman

BESLUT

Nämnden godkänner forskningen på följande villkor.

1. I forskningspersonsinformationen till friska måste syftet med projektet och risker med nasogastrisk tub (aspiration mm) och artärnål anges.
2. Forskningspersonerna ska vårdas på enhet med tillräcklig säkerhet för hantering av artärnål.
3. Friska i beroendeställning ska inte rekryteras.

Hur man överklagar, se särskild information.

Beslut expedierat till behörig företrädare och ansvarig forskare.

Regionala etikprövnings-
nämnden i Stockholm
Inkom: 2016 -01- 19
Dnr: 2016/76-31

Beslutad 2013-03-04

ANSÖKAN OM ETIKPRÖVNING AV FORSKNING SOM AVSER MÄNNISKOR

Information till ansökan, *se Vägledning till ansökan* (*www.epn.se*)
Beroende på vilken forskning som ansökan gäller kommer de uppgifter som efterfrågas att ha olika relevans. Vid ändring av tidigare godkänd ansökan, se *Vägledning till ansökan.*

Till Regionala etikprövningsnämnden i: Stockholm

Den regionala etikprövningsnämnd till vars upptagningsområde forskningshuvudmannen hör, se respektive nämnd *(www.epn.se)*.

Avgift inbetald datum: 13 januari 2016

Observera att en ansökan aldrig är komplett och därmed kan behandlas förrän blanketten är korrekt ifylld och avgiften är betald.

Projekttitel: Helkropps proteinomsättning hos kritiskt sjuka patienter med hypokalorisk och normokalorisk enteral nutrition.

Ange en beskrivande titel på svenska för lekmän. Titeln ska ej innehålla sekretesskyddad information. Ange också i förekommande fall, t.ex. vid klinisk läkemedelsprövning, projektets identitet, forskningsplanens/protokollets nummer, version, datum. Vid ändring av tidigare godkänd ansökan, se Vägledning till ansökan.

Projektnummer/identitet: Version nummer: EudraCT nr (vid läkemedelsprövning):

Uppgifter som fylls i av den regionala etikprövningsnämnden

Ansökan komplett:	Dnr:
Begäran om ytterligare information (i sak):	Begärd information inkommen:
Beslutsdatum:	Expeditionsdatum:

Ansökan avser (gäller även vid begäran om rådgivande yttrande):

Forskning där endast en forskningshuvudman deltar (5 000 kr)	☒
Forskning där mer än en huvudman deltar (16 000 kr)	☐
Forskning där mer än en forskningshuvudman deltar, men där samtliga forskningspersoner eller forskningsobjekt har ett omedelbart samband med endast en av forskningshuvudmännen (5 000 kr)	☐

Endast behandling av personuppgifter (5 000 kr) ☐
(När enbart redan befintliga personregister ska användas, t. ex. nationella databaser)

Forskning som gäller klinisk läkemedelsprövning (16 000 kr) ☒

Ändring av tidigare godkänd ansökan enligt 4 § förordning (2003:615) om etikprövning av forskning som avser människor (2 000 kr) ☐

Om nämnden finner att forskningsprojektet inte faller inom etikprövninglagens tillämpningsområde önskas ett rådgivande yttrande. (Se 4a och 4b §§ i förordning 2003:615 och Vägledning till ansökan)

Ja: ☐ Nej: ☒

1. Information om forskningshuvudman m.m.

1:1 Forskningshuvudman (Se p. 1:1 i Vägledning till ansökan)

Ansökan om etikprövning av forskning ska göras av forskningshuvudmannen. *Med forskningshuvudman avses en statlig myndighet eller en fysisk eller juridisk person i vars verksamhet forskningen utförs.*

Namn: Stockholms Läns Landsting

Adress: Anestesikliniken, Karolinska Universitetssjukhuset Huddinge, 141 86 Stockholm

1:2 Behörig företrädare för forskningshuvudmannen

Behörig företrädare är t.ex. prefekt, enhetschef, verksamhetschef. Forskningshuvudmännen bestämmer själva, genom interna arbets- och delegationsordningar eller genom fullmakt, vem som är behörig att företräda forskningshuvudmannen.

Namn: Patrik Rossi Tjänstetitel: verksamhetschef

Adress: Anestisikliniken, Karolinska Universitetssjukhuset Huddinge, 141 86 Stockholm

1:3 Forskare som är huvudansvarig för genomförandet av projektet (kontaktperson)

(Se p. 9 bil. nr 10 och p. 1:3 i Vägledning till ansökan)

Observera! Den som är huvudansvarig forskare ansvarar för att andra medverkande som ska genomföra projektet har tillräcklig kompetens (vetenskaplig och klinisk) och vid läkemedelsprövning har tillräcklig kunskap om "Good Clinical Practice" (GCP). Vid doktorandstudier är som regel handledaren huvudansvarig forskare.

Namn: Jan Wernerman Tjänstetitel: professor, överläkare

Postadress: Anestesikliniken, Karolinska Universitetssjukhuset Huddinge, 141 86 Stockholm

E-postadress: jan.wernerman@karolinska.se

Telefon: 08-58586395

Mobiltelefon: 070-7461000

1:4 Andra medverkande (Se p. 9 bil. nr 1 och p. 1:4 i Vägledning till ansökan)

Övriga deltagande forskningshuvudmän samt forskare ansvariga för att lokalt genomföra projektet (kontaktpersoner) anges här eller i bilaga med namn och adresser (se p. 9 bil. nr 1).

1:5 Redovisa tillgång till nödvändiga resurser under projektets genomförande

(Se p. 9 bil. nr 9 och p. 1:5 i Vägledning till ansökan)

Ange vem/vilka som har ansvaret (prefekt, verksamhetschef eller motsvarande) för forskningspersonernas säkerhet vid alla enheter/kliniker där forskningspersoner ska delta. Intyg från dessa ansvariga *ska* bifogas (se p. 9 bil. nr 9). Av intyget ska framgå att erforderliga ekonomiska, strukturella och personella resurser finns tillgängliga för att garantera forskningspersonernas säkerhet.

De för projektet nödvändiga resurserna finns inom ALF-anslag samt FoUU-anslag till forskaren.

Se bifogat resursintyg.

1:6 Ansökan/anmälan till andra myndigheter i vissa fall

(se p. 1:6 i Vägledning till ansökan)	Insänd	Datum
a)Vid klinisk läkemedelsprövning: Läkemedelsverket	☐	
b)Vid inrättande av biobank: Socialstyrelsen	☐	
c)Vid undersökning omfattande joniserande strålning: Strålskyddskommitté	☐	

2. Uppgifter om projektet

2:1 Sammanfattande beskrivning av forskningsprojektet

(Se p. 9 bil. nr 2 och p. 2:1 i Vägledning till ansökan)

Beskrivningen ska kunna förstås av nämndens samtliga ledamöter. Undvik därför terminologi som kräver specialkunskaper. Ange bakgrund och syfte för studien samt den/de vetenskapliga frågeställning (ar) som man söker svar på. Ange de viktigaste undersökningsvariablerna. Beskriv vilka kunskapsvinster projektet kan förväntas ge och betydelsen av dessa. Ange om det är en registerstudie, uppdragsforskning etc. För fackmän avsedd detaljerad information om forskningsplan/protokoll (program) *ska* bifogas som bilaga (se p. 9 bil. nr 2). För utformning av forskningsplan/protokoll se p. 2:1 i Vägledning till ansökan. Ange när datainsamlingen beräknas vara avslutad. En utförligare beskrivning av studiens genomförande *avsedd för lekmän* kan vid behov bifogas den för fackmän avsedda obligatoriska forskningsplanen.

Flera författare hävdar att hypokalorisk nutrition är fördelaktig jämfört med full nutrition hos kritiskt sjuka patienter (Arabi et al, Am J Clin Nutr 2011;93:569-577, Krishnan et al, Chest 2003;124:297-305, Marik & Pinsky, ICM 2003;29:867-869). Detta har bl.a resulterat i att rekommendationer för näringstillförsel av kritiskt sjuka patienter skiljer sig åt mellan Europa och Nordamerika. I en tidigare studie fann vi inte något stöd för den hypotesen hos neurokirurgiska patienter med parenteral nutrition (Berg et al, Crit Care 2013;17:R158).

Då jämförde vi full och halv nutrition till neurokirurgiska patienter som erhöll intravenös nutrition på detta sätt. Nutritionen utgjordes av en standardprodukt, och den doserades med hjälp av indirekt kalorimetri. Patienterna erhölla 50% respektive 100% av uppmätt energifrisättning under 24 timmar i randomiserad ordning. Mätningen av helkropps protein kinetik ägde rum under de sista 2 timmarna av respektive nutritionsregim. Resultatet visade en bättre proteinbalans vid tillförsel av 100%, vilket talar till fördel för full nutrition. Emellertid innehöll studien inte någonutvärdering av vårdresultat eller kompliaktioner, vilket kräver en helt annan stolek av studien.

Vi planerar nu att genomföra samma studieprotokoll, men vid tillförsel av enteral nutrition till en blandad grupp av kritiskt sjuka patienter. Tekniken att vädera nutritionsbehandling med helkropps proteinomsättning har inte tidigare använts hos kritiskt sjuka som erhåller enteral nutrition. Därvidlag har vi nyligen gjort ett flertal valideringar av denna teknik vid enteral näringstillförsel (EPN 2011/2029, EPN 2014/116). Resultaten är delvis publicerade och visar då att helkkropps proteinomsättning är relativt konstant under den akuta fasen av kritisk sjukdom, första veckan på intensivvårdsavdelningen (Liebau et al, Crit Care 2015:19:106)

Vi önska nu undersöka effecten av tillförd enteral nutrition av standardtyp i olika mängd på helkropps proteinomsättningen (delstudie A), samt på friska försökpersoner göra en kompletterande studie av upptaget av isotopmärkt aminosyra från tarment (delstudie B, som är en komplettering av EPN 2014/116 ovan)

2:2 Vilken/vilka vetenskaplig (a) frågeställning (ar) ligger till grund för projektets utformning?

Om projektet kan karakteriseras som en hypotesprövning, ange den primära och eventuellt sekundära hypotesen. Hänvisning till mer detaljerad information för fackmän kan ske till bifogad forskningsplan/protokoll enligt punkt 2:1.

Delstudie A. Vilken är effekten av hypokalorisk respektive normokalorisk enteral nutrition av standardtyp på helkropps proteinomsättningen hos kritiskt sjuka patienter?

Delstudie B. Hur överenstämmer upptaget av en isotopmärkt aminosyra med upptaget av proteininnehållet i en standardprodukt för enteral nutrition hos friska individer. Är variabiliteten lika stor hos friska individer som hos intensivvårdspateinter?

2:3 Redogör för resultat från relevanta djurförsök (Gäller klinisk behandlingsforskning)

Om djurförsök inte utförts ange skälen till detta.

Inte relevant med den aktuella frågeställningen.

2:4 Redogör översiktligt för undersökningsprocedur, datainsamling och datas karaktär

(Se p. 9 bil. nr 5 och p. 2:4 i Vägledning till ansökan)

Av beskrivningen ska framgå hur projektet planeras genomföras. Beskriv insamlade datas karaktär. Ange hur datas tillförlitlighet säkerställs (t.ex. kvalitetskontroll/monitorering). Vid enkäter och intervjuer ska beskrivas tillvägagångssätt och t.ex. frågors innehåll och hur slutsatser dras. Enkäter och skattningsskalor *ska* bifogas (se p. 9 bil. nr 5). För medicinsk forskning ska anges t.ex. typer av ingrepp, mätmetoder, antal besök, tidsåtgång vid varje försök, doser och administrationssätt för eventuella läkemedel och/eller isotoper, blodprovsmängd (även ackumulerad mängd vid multipla försök). Ange om och på vilket sätt undersökningsprocedur m.m. skiljer sig från klinisk rutin. Om en behandling studeras för första gången på människa ska detta framgå och relevanta säkerhetsrutiner beskrivas. Ange proceduren för att ge den eventuella behandling efter projektets slut, som kan erfordras. Ange procedur för insamling av biologiskt material. Redogör för datakällor och procedurer vid behandling av personuppgifter. För mer detaljerad information kan hänvisning ske till bilagd forskningsplan.

Delstudie A. Patienter som erhåller full nutrition kan ingå i studien. Om en patient erhåller dialysbehandling skall det noteras och dislysflöden inklusive dragning skall inte ändras under de 4 timmar provtagning sker varje nutritionsdygn och prov från dialysvätskan tas. Patienterna studieras under 2 på varandra följande dygn då de i randomiserad ordning erhåller 50% respektive 100% av uppmätt energifrisättning vid studiens början. Randomisering sker blindat genom dragning av slutna kuvert i grupper om 4. Om möjligt utförs indirekt kalorimetri omedelbart före studien och sedan varje dag under studien.

Tillförseln av 13C-märkt fenylalanin påbörjas 8 timmar före mättillfället för helkropps proteinomsättning. Tiden är vald för att optimera möjligheten till att upptaget är konstant, utan att risken för re-cirkulation av isotopmärkt aminosyra blir onödigt stor. Blodprovstagning sker före start av tillförsel av det 13C-märkta fenylalaninet samt var 10:e minut under den sista halvtimmaen av nutritionsdygnet.

Deuterium-märkt fenylalanin infunderas tillsammans med deuterium-märkt tyrosin, vilket är oxidationsprodukten av fenylalanin. Dessa aminosyror tillförs som primade konstanta infusioner under totalt 180 minuter. Blodprovstagning sker före infusionen samt vid 4 tillfällen under infusionens sista halvtimme.

Vid sidan av mättillfällena innehåller studien inget protokoll. Vid varje provtagningstillfälle insamlas patientdata omfattande patientkaraktäristika, diagnos, pågående nutrition, medcinering, rutinlaboratorieprover, pågående dialys etc genom ett utdrag (genom utskrift) ur den elektroniska patientjournalen.

Bestämning av helkropps proteinomsättningen sker som följer. Infusioner av fenylalanin och tyrosin startas (bolus: 13C-fenylalanin/2H5-fenylalanin 0,5 mg/kg, 2H4-tyrosin 0,15 mg/kg, 2H2-tyrosin 0,3 mg/kg som följs av en kontinuerlig intravenös infusion under cirka fem timmar av 2H5-fenylalanin 0,5 mg/kg/tim, 2H2-tyrosin 0,3 mg/kg/tim, samt en kontinuerlig tillsats till den entrala nutritionen av 13C fenylalanin 0,5 mg/kg/tim). Då steady state uppnås efter 2 timmar utföres mätningar under den påföljande halvtimmen. Blodprov om 4 ml tas vid totalt 10 tillfällen. För patienterna innebär det inga extra kanylsättningar då central venkateter, artärnål och ventrikelslang redan finns hos alla patienter.

Total provtagningsvolym blir vid studie A 80 ml blod.

Delstudie B. Friska försökspersoner av bägge könen kan ingå i studien. Vi försöket börja erhåller försökspersonerna en nutritionssond av tunn modell (sk Clinifeedingsond) genom näsan ned i magsäcken. Därigenom tillförs en standardnutritionsprodukt med pump i en hastighet motsavande 100% av uppskattad basal energifrisättning under 24 timmar. Efter 12 timmar erhåller försökspersonen en intraartiell kanyl för provtagning och samtidigt adderas en tillsats till den entrala nutritionen av 13C fenylalanin 0,5 mg/kg/tim. Därefter sker provtagning var 30:e

minut under 12 timmar, varefter försöket avslutas. Försökpersonerna kan dricka vatten under hela försöket.

Total blodprovstagning blir vid studie B 125 mL blod.

2:5 Redogör för om insamlat biologiskt material kommer att förvaras i en biobank

(Se p. 2:5 i Vägledning till ansökan)

Med biobank avses biologiskt material från en eller flera människor som samlas och bevaras tills vidare eller för en bestämd tid och vars ursprung kan härledas till den eller de människor från vilka materialet härrör. Redogör för var och hur prover som ska sparas förvaras, kodningsprocedurer och villkor för utlämnande av prover. Ange huvudman för biobanken.

Insamlat material lagras i biobank vid Karolinska Universitetssjukhuset Huddinge till dess försöket är publicerat, då kasseras kvarvarande prover. Proverna är hela tiden avidentifierade och endast märkta med nummer. Listan som sammanbinder patientdata med provnumren finns endast på ett ställe hos forskningshuvudmannen.

2:6 Dokumentation, dataskydd och arkivering (Se p. 2:6 i Vägledning till ansökan)

Redogör för hur undersökningsprocedurer och eventuella ingrepp dokumenteras. Ange om band- och videoinspelningar används. Om materialet ska kodas, ange proceduren, vem som förvarar kodlistor/kodnycklar och vem eller vilka som har tillgång till dem, var och hur länge de förvaras samt om materialet kommer att anonymiseras eller förstöras. Redogör för vilken tillgänglighet datamaterialet har och hur det förvaras samt hur erforderligt sekretesskydd erhålls.

Insamlade patientdata och försökspersonsdata samt analysresultat förvaras hos forskaren och lämnar aldrig särskilt låsta utrymmen på sjukhuset, innan de är avidentifierade. Materialet kommer helt att avidentifieras när sammanställningen och publicering är klar.

2:7 Redogör för tidigare erfarenheter (egna och/eller andras) av den använda proceduren, tekniken eller behandlingen

Särskilt angeläget är att redovisning av risker för komplikationer görs tydliga och i förekommande fall med angivande av relevanta publikationer. Vid nya behandlingar av patienter, t.ex. med läkemedel, bör anges hur många patienter (med aktuell eller annan åkomma) som tidigare erhållit föreslagen behandling, läkemedelsdosering (eller annan dosering) samt hur långa behandlingsperioder som studerats.

Undersökningen är en forsättnig av studierna med Dnr 290/03, 2006-1499, 2009-1647, 2011-2029, 2014-116. Forskningshuvudmannen har stor erfarenhet av metaboliska studier av kritiskt sjuka patienter och försökspersoner.

3. Uppgifter om forskningspersoner

3:1 Hur görs urvalet av forskningspersoner? (Se p. 9 bil. nr 3 och p. 3:1 i Vägledning till ansökan)

Med forskningsperson avses en levande människa som forskningen avser. Ange urvalskriterier (inklusion och exklusion). Redogör för på vilket sätt forskaren kommer i kontakt med/får kännedom om lämpliga forskningspersoner. Om annonsering sker, *ska* annonsmaterialet insändas som bilaga (se p. 9 bil. nr 3). Om t.ex. barn eller personer som tillfälligt eller permanent inte är kapabla att ge ett eget informerat samtycke ska ingå i projektet, ska detta särskilt motiveras. Om vissa grupper utesluts från deltagande i projektet ska detta särskilt motiveras.

Som forskningspersoner patienter rekryteras patienter på intenisvvårdsavdelninén som är cirkulatoriskt stabila samt som erhåller full enteral nutrition, de friska försökpersonerna rekryteras bland individer som spontant anmält intresse att delta i metaboliska studier

3:2 Ange relationen mellan forskare/försöksledare och forskningspersonerna

- [x] Behandlare (t.ex. läkare, psykolog, sjukgymnast) - forskningsperson (t.ex. patient, klient)
- [ ] Kursgivare (lärare) - student
- [ ] Arbetsgivare - anställd
- [ ] Annan relation som kan tänkas medföra risk för påverkan. Beskriv:

3:3 Redogör för det statistiska underlaget för studiepopulationens (-ernas)/ undersökningsmaterialets (-ens) storlek (Se p. 3:3 i Vägledning till ansökan)

Redovisa statistisk styrka, så kallad "power"- beräkning eller redovisa motsvarande överväganden som tydliggör studiens möjligheter att besvara frågeställningarna.

Studie A. Någon skillnad mellan att erhålla 50% av nutritionen före eller efter 100% av nutritionen sågs ej i den tidigare studien, därför förväntas inte heller någon sådan skillnad i denna studie. Patienterna blir sina egna kontroller. Därför bedöms att resultaten kan bli konklusiva om minst 6 evaluerbara patienter finns i vardera gruppen. Totalt kommer det att finnas 12 patienter, som kommer att jämföras med sig själva. Om de kan betraktas som en grupp kommer en skillnad av uppmätta variabler om 75% av standarddeviationen att kunna upptäckas.

Studie B. Resultaten av patientstudien (EPN 2014/116) med n=10 visade en relativt hög variabilitet över tiden som var interindividuell. Huvudsyftet är nu att undersöka om detta är fysiologist, dvs även uppträder hos friska unders samma nutirtionsbetingelse, eller om det relaterar till kritisk sjukdom. Av detta skäl blir valet n=10 evaluerbara försökspersoner. Om resultatet av något skäl inte blir konklusivt med n=10, får vi inkomma med kompletterande EPN ansökan.

3:4 Kan forskningspersonerna komma att inkluderas i flera studier samtidigt eller i nära anslutning till denna? I så fall, vilken typ av forskning?

(Se p. 3:4 i Vägledning till ansökan)

Det finns inget hinder att dessa forskningspatienter ingår i andra studier. Försökspersonerna skall inte ha deltagit i andra studier under 1 månad före studien.

3:5 Vilket försäkringsskydd finns för de forskningspersoner som deltar i projektet?

Det åligger forskningshuvudmannen att kontrollera att det finns försäkring som täcker eventuella skador som kan uppkomma i samband med forskningen.

Patienterna ochförökspersoneran omfattas av patientförsäkringen.

3:6 Vilken ekonomisk ersättning eller andra förmåner utgår till de forskningspersoner som deltar i projektet och när betalas ersättningen ut? Utförligare beskrivning kan lämnas i bilaga.

(Se p. 9 bil. nr 11 och p. 3:6 i Vägledning till ansökan)

Ersättning för obehag och besvär. Ange belopp (före skatt):	SEK 3000 (studie B)	
Ersättning för förlorad arbetsinkomst	☐ Ja	☐ Nej
Reseersättning	☐ Ja	☐ Nej
Befrielse från kostnader för läkemedel	☐ Ja	☐ Nej

Befrielse från andra kostnader. Vilka?

Andra förmåner. Vilka?

När betalas ersättningen ut?

Ingen ersättning betalas ut ☒

4. Information och samtycke

4:1 Proceduren för och innehållet i den *information* som lämnas då forskningspersoner tillfrågas om deltagande

(Se p. 9 bil. nr 4 och Vägledning till forskningspersonsinformation.)

Enligt 16 § lag (2003:460) om etikprövning av forskning som avser människor ska forskningspersonen informeras om den övergripande planen för forskningen, syftet med forskningen, de metoder som kommer att användas, de följder och risker som forskningen kan medföra, vem som är forskningshuvudman, att deltagande i forskningen är frivilligt och forskningspersonernas rätt att när som helst avbryta sin medverkan. Beskriv hur och när information ges och vad den innehåller. Ange vem som informerar. Normalt ska en kortfattad och lättförståelig skriftlig information ges. Denna skriftliga information *ska* bifogas ansökan (se p. 9 bil. nr 4). Om ingen eller ofullständig information ges, måste skälen för detta noggrant anges.

Forskningspatienter eller anförvant informeras och tillfrågas muntligt och skriftligt om deltagande i studie A. Se bifogad patientinformation.

Försökspersoner informeras och tillfrågas muntligt och skriftligt om deltagande i studie B. Se bifogad försökpersonsinformation.

4:2 Hur och från vem inhämtas *samtycke*? (Se Vägledning till forskningspersonsinformation)

Beskriv proceduren; vem som frågar, när detta sker och hur samtycket dokumenteras. Utförlig redovisning är särskilt viktig då barn eller personer med nedsatt beslutskompetens ingår i studien, likaså vid studier av en grupp/grupper, t.ex. skolklasser, föreningar, organisationer, företag, kyrkosamfund, församlingar eller grupper som interagerar inom sociala medier.

Se 4:1

5. Forskningsetiska överväganden

5:1 Redogör för alla risker som deltagandet kan medföra

Dessa kan vara t.ex. fysisk eller psykisk skada, smärta, obehag eller integritetsintrång på kort eller lång sikt. Ange vilka åtgärder som har vidtagits för att förebygga riskerna som nämns ovan samt vilken beredskap som finns för att hantera sådana komplikationer. Ange vilka/de metoder som kommer att användas för att efterforska, registrera och rapportera oönskade händelser.

Tillförsel av isotopmärkt aminosyra intravenöst är inte förenat med några som helst risker.

Patienterna i delstudie A har rutinmässigt ven- och artärkatetrar så någon ökad risk för insättande av såna föreligger inte.

Vidare kommer patienterna i delstudie A att under 24 timmar endast erhålla 50% av nutritionen, vilket skiljer sig från rutinen vid Karolinska Huddinge, men vilket är rutin vid många sjukhus i andra delar av världen, t.ex nordamerika.

Försökspersonerna i delstudie B kommer att erhålla en nasogastrisk sond av tunn typ samt en artärkateter för provtgning. Båda procedurerna är rutin inom sjukvården som i studien utförs av vana personer. Lokalbedövning ges vid anbringandet av artärkateter. I båda fallen föreligger obehag, men inte smärta, och risken är ringa.

I delstudie A kommer den totala blodprovstagning att understigande 80 ml, och i delstudie B 125 ml vilket i bägge fallen endast medför en ringa risk.

5:2 Redogör för möjlig nytta för de forskningspersoner som ingår i projektet (gäller särskilt behandlingsforskning)

Forskningspersonerna har ingen förutsägbar personlig nytta av att delta i studierna.

5:3 Identifiera och precisera om eventuella etiska problem (fördelar/nackdelar) kan uppstå i ett vidare perspektiv genom projektet (Se p. 5:3 i Vägledning till ansökan)

Här kan redovisas om exempelvis vissa grupper (andra än de forskningspersoner som ingår i forskningsprojektet) kan komma att utpekas/få hjälp som ett resultat av studien.

Nyttan med projektet, dvs den kunskapsvinst som erhålls är stor för gruppen kritiskt sjuka spatienter. Det saknas idag adekvat metodik för att utvärdera hur nutrition ska doseras hos de akut sjuka intensivvårdspatienterna. Utvidgningen och slutförandet av den aktuella studien innebär värdefulla steg för att vinna denna kunskap. Därför blir det i ett allmänt perspektiv vinsten stor i förhållande till de risker som deltagande försökspersoner och patienter utsätts för och det intrång som görs i deras integritet.

6. Redovisning av resultaten

6:1 Hur garanteras forskningshuvudmannen och medverkande forskare tillgång till data (anges vid t.ex. uppdragsforskning) och vem ansvarar för databearbetning och rapportskrivning?
(Se p. 6:1 i Vägledning till ansökan)

Ej applicerbart.

6:2 Hur kommer resultaten att göras offentligt tillgängliga? Kommer studien att insändas för publicering i tidskrift eller publiceras på annat sätt? (Se p. 6:2 i Vägledning till ansökan)

Ange i vilken form resultaten planeras offentliggöras samt tidsplan för detta.

Resultaten avser att publiceras i vetenskaplig tidskrift.

6:3 På vilket sätt garanteras forskningspersonernas rätt till integritet när materialet offentliggörs/publiceras?

Redovisas resultat på statistisk gruppnivå? Beskriv procedurer eller metoder för avidentifiering/anonymisering.

Patienterna och försökspersonerna kommer inte att kunna identifieras i det publicerade materialet.

7. Redovisning av ekonomiska förhållanden och beroendeförhållanden

Redovisning enligt punkterna 7:1-7:3 syftar till att tydliggöra alla direkta eller indirekta förhållanden, som kan tänkas påverka forskarens relation till forskningspersonerna (vid t.ex. informations-, samtyckes-, genomförandeprocedurer).

7:1 Vid uppdragsforskning

Ange uppdragsgivaren t.ex. ett företag (vid klinisk läkemedelsprövning eller prövning av andra nya produkter), en organisation eller en myndighet.

Namn: Kontaktperson:

Adress: Telefon/mobiltelefon:

Ange uppdragsgivarens relation till forskningshuvudmannen/medverkande forskare, t.ex. anställningsförhållande

7:2 Redovisa eventuella ekonomiska överenskommelser med uppdragsgivare eller andra finansiärer (namn, belopp)

Vid klinisk läkemedelsprövning bör hänvisning ske till ingånget avtal med sjukvårdshuvudmannen. Liknande överenskommelser kan förekomma vid annan uppdragsforskning och bör redovisas på samma sätt. Separata överenskommelser med den/de som ska genomföra forskningen ska redovisas. Belopp som kommer att erhållas för studien/ersättning till kliniken/genomföraren, vad ersättningen bör täcka och ev. belopp som erhålls per forskningsperson, bör också anges här.

Ej applicerbart

7:3 Redovisa forskningshuvudmannens, huvudansvarig forskares och medverkande forskares egna intressen

Här redovisas t.ex. aktieinnehav, anställning, konsultuppdrag i finansierande företag, eget företag som kan få (direkt eller indirekt) ekonomisk vinst av forskningen.

Ej applicerbart

8. Undertecknande

Behörig företrädare för sökande forskningshuvudman enligt p. 1:2.

Ort: Stockholm Datum: 13/1 2016

Signatur:

Namnförtydligande: Patrik Rossi

Tjänstetitel: verksamhetschef

Undertecknad forskare som genomför projektet (kontaktperson) enligt p. 1:3 intygar härmed att forskningen kommer att genomföras i enlighet med ansökan.

Ort: Stockholm Datum: 13/1 2016

Signatur:

Namnförtydligande: Jan Wernerman

Tjänstetitel: professor, överläkare

9. Förteckning över bilagor (Se p. 9 i Vägledning till ansökan)

Dokument som, i tillämpliga fall, ska bifogas *om inte motsvarande information finns i blanketten* har markerats med x. Markera de bilagor som skickas in med denna ansökan.

Insänd med ansökan	Bil nr	Beskrivning	Klinisk läkemedels-prövning	Annan forskning
☐	1	Deltagande forskningshuvudmän och medverkande forskare (kontaktpersoner) vid forskning där mer än en forskningshuvudman deltar. Se p. 1:4	x	x
☒	2	För fackmän avsedd forskningsplan, vid behov även för lekmän avsedd bilaga. Se p. 2:1 och Vägledning till forskningsplan/forskningsprotokoll (program)	x	x
☐	3	Annonsmaterial för rekrytering av forskningspersoner. Se p. 3:1 och i Vägledning till ansökan p. 3:1	x	x
☒	4	Skriftlig information till dem som tillfrågas. Se p. 4:1 och Vägledning till forskningspersonsinformation och (i förekommande fall) separat samtyckesformulär	x	x
☐	5	Enkät, frågeformulär. Se p. 2:4	x	x
☐	6	Gemensam EU blankett (gäller fr.o.m. den 1 maj 2004), gäller även vid ändring. För information se Läkemedelsverkets hemsida, www.lakemedelsverket.se	x	
☐	7	Sammanfattning av protokollet på svenska	x	
☐	8	Prövarhandbok alt. bipacksedel/produktresumé/IB	x	
☒	9	Intyg från verksamhetschef/motsv. om resurser för forskningspersonernas säkerhet. Se p. 1:5 och förslag till utformning av resursintyg i Vägledning till ansökan p. 1:5	x	x
☒	10	CV för forskare (samma som p. 1:3) med huvudansvar för genomförandet, redovisa forskarens (- arnas) kompetens av relevans för studien. Se Vägledning till ansökan p. 1:3	x	x
☐	11	Beskrivning av ersättning till forskningspersoner. Se p. 3:6 och i Vägledning till ansökan p. 3:6	x	x

Övriga bilagor som bifogas ansökan:

Bilaga 4

2016/76-31

FÖRSÖKSPERSONSINFORMATION

Angående undersökning av näringsupptaget vid sondmatning.

Du har anmält Ditt intresse för deltagande i en studie där upptaget av aminosyror från proteiner i sondmat studeras. Resultaten förväntas leda till att vi kan utveckla bättre metodik att studera proteinomsättningen hos svårt sjuka människor och hur de förmår att tillgodogöra sig tillförd näring.

Undersökningen pågår i 24 timmar. Under två dagar före försöket får Du inte ta några läkemedel och Du bör i övrigt hålla Dig till regelbundna vanor och normal kost samt undvika alkohol. Försöket inleds med att Du kommer till sjukhuset vid 18-tiden och då erhåller Du en tunn slang via näsan ner i magsäcken. Genom den tillförs sedan sondmat under 24 timmar. Under försöket kommer Du att kunna dricka kranvatten i måttlig mängd. Du övernattar på sjukhuset. Nästa morgon sker en tillsats av isotopmärkt aminosyra till sondmaten och blodprovstagning sker genom en kvarliggande artärkateter var 30 minut totalt 25 prover på 12 timmar. I och med att försöket är avslutat på kvällen dag 2 finns inga ytterligare restriktioner relaterade till försöket.

Risker och obehag under försöket är fokuserade till magsonden och artärkatetern. Bedövningsgel ges i näsan vid införandet av slangen och på plast fördras magsonden väl. Lokalbedövning av huden sker innan artärkatetern anlägges samt ordentligt tryck och högläge vid borttagandet. Tillförsel av sondmat och isotopmärkt aminosyra är inte förenade med några obehag eller risker. Den arteriella kanylen, magssonden sjukhusvistelse i 1 dygn innebär självklart ett integritetsintrång under försöket.

Deltagandet i försöket är helt frivilligt och Du kan när som helst utan särskild motivering avbryta Din medverkan. Ersättning för förlorad arbetsinkomst om 3.000 kr ges, denna ersättning är beskattningsbar inkomst.

Insamlade personuppgifter lyder under personuppgiftslagen (PuL 1998-204). De uppgifter som insamlas i studien kommer endast att hanteras av undersökaren och de kommer att avidentifieras då studien publicerats. Som deltagare i studien kan Du begära att få veta vilka uppgifter som registrerats, vilket är möjligt fram till att uppgifterna avidentifieras. Undertecknad är personuppgiftsansvarig.

Hanteringen av de prover som tas i studien lyder under Biobankslagen (BbL 2002:297). Proverna är endast märkta med ett nummer och listan som identifierar proverna med en individ finns endast hos undersökaren. Denna lista förstörs och resterna av proverna kasseras då studien publicerats. Då proverna sparas i väntan på publicering förvaras de i en biobank. Om dessa prover skulle användas till andra analyser än vad som angivits i denna patientinformation måste Etikprovningsnämnden ge sitt godkännande. Du kan då komma att kontaktas igen för nytt samtycke. Du kan när som helst och utan närmare motivering begära att de prover som donerats av Dig och som finns i biobanken skall förstöras och avidentifieras.

Jan Wernerman
Professor
Anestesi- och intensivvårdsklin
Tfn: 08 585 863 95

Samtycke

Jag har erhållit muntlig och skriftlig information om studien ”näringsupptaget vid sondmatning”, och jag

………………………………….. (namn klarskrift)

ger mitt medgivade till deltagande i studien.

………………………………….. (datum)

………………………………….. (namnteckning)

………………………………….. (namn klarskrift)

Bilaga 4

Patientinformation

Du har tillfrågats om Ditt deltagande i studien "Effekten av näringstillförsel på proteinomsättningen hos patienter inom intensivvården".

Det är väl känt att patienter inom intensivvården måste erhålla näringstillförsel. Det dock oklart hur stor tillförseln av näring bör vara. I denna studie undersöks energiförbrukningen samt proteinomsättningen vid 2 olika nivåer av tillförsel, vilket kommer att kunna ge svar på vilken tillförsel som är optimal.

Deltagande i försöket innebär en studie under 48 timmar där olika nivåer av näringstillförsel under vardera 24 timmar jämförs. En aminosyra märkt med stabil, icke-radioaktiv isotop tillförs, och viss extra blodprovstagning äger rum. En något lägre näringstillförsel under 24 timmar är ofarlig. Tillförsel av en liten mängd isoptopmärkt aminosyra är också helt ofarlig Blodprovstagningen sker via befintlig artärkateter. Den totala blodprovtagningsvolymen i denna studie uppgår till maximalt 8 cl. Studien innehåller två perioder med olika nivå av näringstillförsel. Insamlade data kommer att avidentifieras så snart studien är avslutad.

Du har som patient ingen direkt fördel att delta i denna studie. Indirekt kommer dock övervakningen av ämnesomsättningen att bli mer intensiv, vilket ger möjlighet att bättre dosera nutritionen. Resultatet av studien kommer att vara till hjälp för patienter i samma situation i framtiden. Som en del av kvalitetskontrollen av studien kan medarbetare frånsjukhusets monitoreringsenhet eller från statlig myndighet komma att ta del av journalhandlingarna. Detta sker givetvis under tystnadsplikt.

Deltagandet i studien är helt frivilligt och Du kan när som helst utan särskild motivering avbryta Ditt deltagande i studien.

Insamlade personuppgifter lyder under personuppgiftslagen (PuL 1998-204). De uppgifter som insamlas i studien kommer endast att hanteras av undersökaren och de kommer att avidentifieras då studien publicerats. Som deltagare i studien kan Du begära att få veta vilka uppgifter som registrerats, vilket är möjligt fram till att uppgifterna avidentifieras. Undertecknad är personuppgiftsansvarig.

Hanteringen av de prover som tas i studien lyder under Biobankslagen (BbL 2002:297). Proverna är endast märkta med ett nummer och listan som identifierar proverna med en individ finns endast hos undersökaren. Denna lista förstörs och resterna av proverna kasseras då studien publicerats. Då proverna sparas i väntan på publicering förvaras de i en biobank. Om dessa prover skulle användas till andra analyser än vad som angivits i denna patientinformation måste Etikprovningsnämnden ge sitt godkännande. Du kan då komma att kontaktas igen för nytt samtycke. Du kan när som helst och utan närmare motivering begära att de prover som donerats av Dig och som finns i biobanken skall förstöras och avidentifieras.

Jan Wernerman
Professor
Anestesi- och intensivvårdsklin
Tfn: 08 585 863 95

Samtycke (från patient)

Jag har erhållit muntlig och skriftlig information om studien ”Effekten av näringstillförsel på proteinomsättningen hos patienter inom intensivvården”, och jag ger mitt medgivade att deltaga i studien.

…………………………………….. (datum)

…………………………………….. (namnteckning)

…………………………………….. (namn klarskrift)

Bilaga 4

Patientinformation (till anhörig)

Du har tillfrågats om Din anhörigs deltagande i studien ”Effekten av näringstillförsel på proteinomsättningen hos patienter inom intensivvården”.

Det är väl känt att patienter inom intensivvården måste erhålla näringstillförsel. Det dock oklart hur stor tillförseln av näring bör vara. I denna studie undersöks energiförbrukningen samt proteinomsättningen vid 2 olika nivåer av tillförsel, vilket kommer att kunna ge svar på vilken tillförsel som är optimal.

Deltagande i försöket innebär en studie under 48 timmar där olika nivåer av näringstillförsel under vardera 24 timmar jämförs. En aminosyra märkt med stabil, icke-radioaktiv isotop tillförs, och viss extra blodprovstagning äger rum. En något lägre näringstillförsel under 24 timmar är ofarlig. Tillförsel av en liten mängd isoptopmärkt aminosyra är också helt ofarlig Blodprovstagningen sker via befintlig artärkateter. Den totala blodprovtagningsvolymen i denna studie uppgår till maximalt 8 cl. Studien innehåller två perioder med olika nivå av näringstillförsel. Insamlade data kommer att avidentifieras så snart studien är avslutad.

Din anhörig har som patient ingen direkt fördel att delta i denna studie. Indirekt kommer dock övervakningen av ämnesomsättningen att bli mer intensiv, vilket ger möjlighet att bättre dosera nutritionen. Resultatet av studien kommer att vara till hjälp för patienter i samma situation i framtiden. Som en del av kvalitetskontrollen av studien kan medarbetare frånsjukhusets monitoreringsenhet eller från statlig myndighet komma att ta del av journalhandlingarna. Detta sker givetvis under tystnadsplikt.

Deltagandet i studien är helt frivilligt och Du eller Din anhörig kan när som helst utan särskild motivering avbryta Din anhörigs deltagande i studien utan att det påverkar våden i övrigt på något säatt..

Insamlade personuppgifter lyder under personuppgiftslagen (PuL 1998-204). De uppgifter som insamlas i studien kommer endast att hanteras av undersökaren och de kommer att avidentifieras då studien publicerats. Som deltagare i studien kan Din anhörig begära att få veta vilka uppgifter som registrerats, vilket är möjligt fram till att uppgifterna avidentifieras. Undertecknad är personuppgiftsansvarig.

Hanteringen av de prover som tas i studien lyder under Biobankslagen (BbL 2002:297). Proverna är endast märkta med ett nummer och listan som identifierar proverna med en individ finns endast hos undersökaren. Denna lista förstörs och resterna av proverna kasseras då studien publicerats. Då proverna sparas i väntan på publicering förvaras de i en biobank. Om dessa prover skulle användas till andra analyser än vad som angivits i denna patientinformation måste Etikprovningsnämnden ge sitt godkännande. Du kan då komma att kontaktas igen för nytt samtycke. Din anhörig kan när som helst och utan närmare motivering begära att de prover som donerats av honom/henne och som finns i biobanken skall förstöras och avidentifieras.

Jan Wernerman
Professor
Anestesi- och intensivvårdskliniken
Tfn: 08 585 863 95

Samtycke (från anhörig)

Jag har erhållit muntlig och skriftlig information om studien ”Effekten av näringstillförsel på proteinomsättningen hos patienter inom intensivvården”, och jag ger mitt medgivade att min anhörig

………………………………….. (personnummer)

………………………………….. (namn klarskrift)

deltar i studien.

………………………………….. (datum)

………………………………….. (namnteckning)

………………………………….. (namn klarskrift)

Bilaga 9

2016/76-31

Resursintyg

Avseende studien ”Helkropps proteinomsättning hos kritiskt sjuka patienter med hypokalorisk och normokalorisk enteral nutrition”.

Erforderliga personella och ekonomiska resurser för ovanstående projekt finns dels inom ramen för ALF-anslag till sökanden samt inom FOUU-budgeten vid anestesikliniken Karolinska Huddinge.

Karolinska Huddinge 13 januari 2016

Jan Wernerman
Sökande

Patrik Rossi
verksamhetschef
Anestesi- och Intensivvårdskliniken
Karolinska Huddinge

Bilaga 10

2016/76-31

CURRICULUM VITAE

Name	Wernerman Jan
Date of birth	August 30, 1949
Address	Department of Anaesthesia and Intensive Care<br>Karolinska University Hospital, Huddinge<br>Karolinska Institutet<br>S-141 86 Stockholm, Sweden
Telephone hospital	+46-8-58 58 63 95
Fax	+46-8-779 54 24
E-mail	jan.wernerman@karolinska.se
Telephone/fax home	+46-8-26 80 27
Mobile	+46-70-746 10 00

Education

1974	Graduate in medicine
1977	Bachelor of medicine
1979	M.D. licensed doctor
1983	Specialist in Anaesthesiology & Intensive Care Medicine
1985	Ph.D. Anaesthesiology & Intensive Care Medicine
1988	Ass Prof of Anaesthesiology & Intensive Care Medicine
2000	Professor of Anaesthesiology & Intensive Care Medicine

Academic credentials

Head supervisor of 12 PhD students

> 200 original publications

Present funding

Recommended grant from Medical Research Council renewed since 1994.
Renewed local SLL grant since 2004.
Project associated grants from industry.
Working group annual budget approx 2.5 MSEK.

Former appointments

Chairman at the Department of the Anaesthesiology and Intensive Care Unit, Huddinge University Hospital 1994-2000

Chairman at the Department of the Anaesthesiology and Intensive Care Unit, St Görans's Hospital Stockholm 1990-1994

Director Department of Intensive Care, Huddinge University Hospital 1987-1990

Chairman ESPEN Scientific Committee 1995-1998, Member of ESPEN Scientific Committee 1992-1995

Chairman of the Working Group for Intensive Care in the Swedish Society for Anaesthesiology & Intensive Care 1995-1998.

Director for the Division of Anesthesiology in the KARO Institution, Karolinsksa Institutet 1994-2001

Member of the ESICM Executive Committee and Treasurer of the society 2002-2007

President of the Swedish Association for Anesthesia and Intensive Care Medicine (SFAI) 2007-2010

Member of the Board of the Scandinavian Society for Anesthesiology and Intensive Care Medicine (SSAI) 2008-2011.

Section head Metabolism, Endocinology and Nutrition, member of Coucil, member of Congress Committee ESICM since 2009-2012.

Present appointments

Professor of Anaesthesiology and Intensive Care at Karolinska Institutet since 2000, senior professor since 2014

Director of the Unit of Anaesthesiologic Metabolism at the Clinical Research Centre, Karolinska University Hospital, Huddinge at Karolinska Institutet, Stockholm since 1995.

Member of the Steering Group for Post-graduate Intensivist training in Scandinavia since 1998, and member of the Organising Committee for the biannual Scandinavian Intensivist Meeting since 2014 .

Treasurer of the Swedish Association for Anesthesia and Intensive Care Medicine (SFAI) 2015-
Associate Editor of *Acta Anaesthesiologica Scandinavia* since 1998.

Member of the Editorial Boards of *Clinical Nutrition*, *Journal of Parenteral and Enteral Nutrition*, *Current Opinion in Clinical Nutrition and Metabolic Care* and *Critical Care.*

Hej,

Tacksam för hantering
Betalning är på väg. (se order)

Hälsn. [illegible]

0737-127393

Karolinska Institutet

UTBETALNINGSORDER

Betalningsavsändare	Betalningsmottagare
**Enhet**<br>Anestesi	**Regionala Etikprövningsnämnden Stockholm**

Ärende / Text

Forskarens namn: Jan Wernerman

Projekttitel: Helkropps proteinomsättning hos kritiskt sjuka patienter med hypokalorisk och normokalorisk enteral nutrition

Avgift kr: 5 000 kr

Enligt beslut	Bifogar bilaga
	Ja

Namn

Datum 2016-01-13

Lev nr	Verifikationsnummer
**2111016489**	

Bankgironr.	Text till leverantör	
**794-5496**	Jan Wernerman	0

Förfallodag	Verifikationstext	
	Jan Wernerman	0

Konto	Proj	Fin	Mpt	Belopp Deb.	Belopp Kred.
	**H95015133**		**EPS**		**5 000,00**

Attest Utbet.	Kontering	Bokförd datum
[signature] Sigridur Kalman		

[signature]

Forskningsplan 2016-01-11

Helkropps proteinomsättning hos kritiskt sjuka patienter normokalorisk och hypokalorisk enteral nutrition.

Regionala etikprövnings-
nämnden i Stockholm
Inkom: 2016 -01- 29
Dnr: 2016/76-3

Ansvarig undersökare: Jan Wernerman

Bakgrund

Kroppens proteinomsättning och effekten av nutrition har traditionellt bestämts med hjälp av kvävebalans. Det är en teknik med många begränsningar, som blir särskilt tydliga då bestämningar skall göras under omständigheter då individen inte är adapterad till nivån på vare sig den kaloritillförsel eller den proteintillförsel som skall värderas. Kvävebalans ger tillförlitliga resultat då sammansättning av kaloritillförsel eller proteintillförsel skall värderas vid isokalorisk och isonitrogen kost som individen är fullt adapterad till.

Hos sjuka individer saknas emellertid de förutsättningar som är nödvändiga för ett meningsfullt användande av kvävebalans. Inte desto mindre vilar de rekommendationer som idag finns vad beträffar främst proteintillförsel till sjuka individer till en stor del på kvävebalansstudier.

Ett annat tänkbart tillvägagångssätt är att göra bestämningar av proteininnehållet i kroppen. Tillgängliga metoder är bl.a magnetresonans, ultraljud och datortomografi. Den tekniska utvecklingen bland dessa metoder gör att man i framtiden sannolikt kommer att kunna bestämma även små skillnader. En annan svårighet som sannolikt även den kommer att kunna bättre bemästras i framtiden, är det variabla vatteninnehållet i vävnaderna.

Den mest lovande tekniken idag är att bestämma helkropps proteinkinetik med hjälp av en isotopmärkt aminosyra. Tekniken ger möjlighet att differentiera mellan syntes, degradation och oxidation, vilket ingen av övriga tekniker förmår. Tekniken kan dessutom genom blodkärlskateteriseringar över specifika vävnadsområden och/eller vävnadsbiopsier utvidgas till att även omfatta samma bestämningar på vävnadsnivå. I sin enklaste form innebär tekniken att en isotopmärkt aminosyra injiceras, varefter sekvensiell provtagning sker under en ekvilibreringsperiod. Beroende på val av isotopmärkning kan insamlig av CO_2 i utandningsluft för analys vara aktuell för att kunna bestämma oxidationen.

Den mest tydliga begränsningen i den enkla formen av denna teknik är att endast de vävnader vars aminosyraomsättningen ekvilibrerar med provtagnings-poolen kommer att ingå i mätningen

I det postabsortiva tillståndet ger detta full information. Likaså då en individ erhåller parenteral nutrition men konstant intravenös tillförsel under mätperioden [1]. Då en individ erhåller enteral nutrition kompliceras förutsättningarna. Även då den enterala nutritionen tillförs med konstant hastighet, är inte detta liktydigt med en konstant tillförsel till provtagnings-poolen (normalt blodplasma).

Vi har kunnat visa att efter 6 timmar av konstant tillförsel av ett isotopinmärkt mjölkprotein finns ett konstant inflöde av den isotopmärkta aminosyran till provtagnings-poolen [2]. Detta försök gjordes med en relativt liten fraktion av det totala näringsbehovet. Hos såväl friska försökspersoner som hos IVA patienter kunde ett sådant tillstånd av konstant inflöde dokumenteras. Emellertid var extraktionen i splanknikusområdet påtagligt hög och den var olika för friska försökspersoner och för IVA patienter (figur 1).

Figur 1.

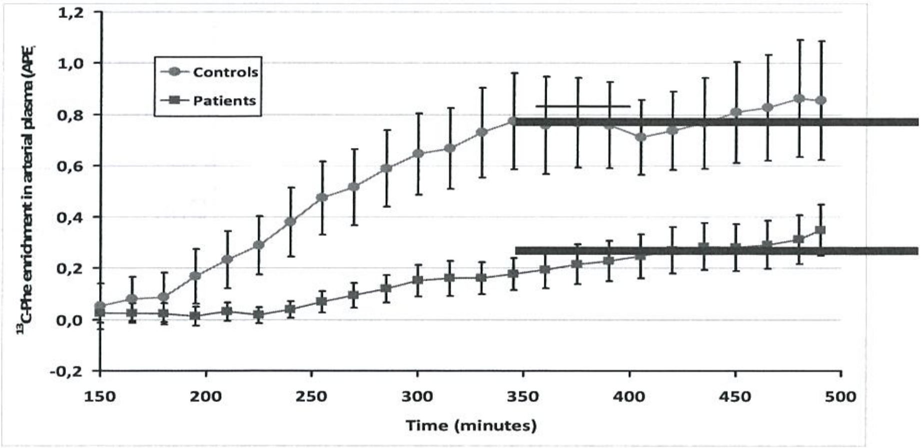

Liebau et al, Am J Clin Nutr 2015, 101:549-57

Vi har dessutom kunnat visa att hastigheten på inflödet till provtagnings-poolen av en fri isotopmärkt aminosyra given samtidigt med mjölkprotein och den däruti inbyggda isotopmärkta aminosyran är detsamma efter en stabiliseringperiod (figur 2) [3]. Detta betyder att vi i de fortsatta försöken inte behöver vara hänvisade till särskilda proteiner med inbyggda isotopmärkta aminosyror, utan kan använda standardnutrition med en parallell tillförsel av fri isotopmärkt aminosyra.

Figur 2.

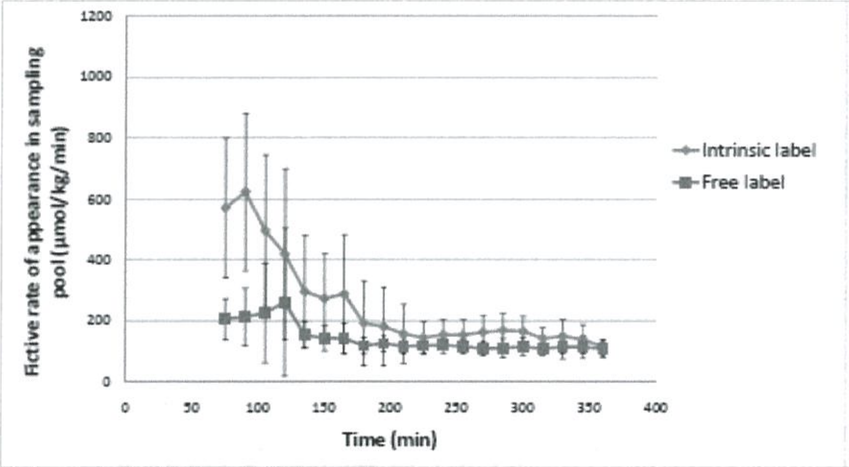

Liebau et al Crit Care 2015,19:116

I de pilotförsök som refererats ovan användes en relativt låg tillförsel av enteral nutrition varvid en relativt hög splanknisk extraktion erhölls. Det är inte självklart att detta även gäller för full nutrition. En validering av detta är utfört som ett pilotförsök (EPN 2014/116). Resultatet visar en påtaglig variabilitet över tiden hos de kritiskt sjuka patienterna. Det är oklar om detta är fysiologiskt, dvs även förkommer hos friska individer under motsvarande omständigheter, eller om det endast förekommer vid kritisk sjukdom.

I annan tidigare studie har vi jämfört full och halv nutrition till neurokirurgiska patienter som erhöll parenteral nutrition på detta sätt [4]. Nutritionen utgjordes av en standardprodukt, och

den doserades med hjälp av energifrisättningen mätt med indirekt kalorimetri, MEE, och patienterna randomiserades till att erhålla 50% av MEE under 24 timmar före 100% av MEE under 24 timmar, eller tvärtom. Mätningen av helkropps protein kinetik ägde rum under de sista 2 timmarna av respektive nutritionsregim. Resultatet visar att proteinbalansen är gynnsammare tillförsel av 100% av MEE, men att oxidationen inte skiljer sig åt. Detta demonstrerar att vid en proteintillförsel om 1,1 g/kg och en energitillförsel på nivån av EE, sker ingen oxidation av överflödigt tillfört protein.

Skälet att genomföra den studien var att ett antal författare hävdat att hypokalorisk nutrition är fördelaktig jämfört med full nutrition hos kritiskt sjuka [5-7]. I vår studie fann vi inte något stöd för den hypotesen hos neurokirurgiska patienter med parenteral nutrition. Resultatet visade en bättre proteinbalans vid tillförsel av 100%, vilket talar till fördel för full nutrition. Emellertid innehöll studien inte någon utvärdering av vårdresultat eller komplikationer, vilket skulle ha krävt en helt annan stolek av studien.

Vi planerar nu att i delstudie A genomföra samma studieprotokoll som hos de neurokirurgiska patienterna, men vid tillförsel av enteral nutrition. Frågeställningarna är desamma som i det tidigare försöket, med tillägg av, vilken är splanknikus extraktionen vid respektive tillförselnivå? I delstudie B vill vi undersöka om friska individer uppvisar samma variabilitet över tiden som kritiskt sjuka individer vid tillförsel av en isotopmärkt aminosyra parallellt med full enteral nutrition.

Metodik

Upptaget och utnyttjandet av enteral nutrition bestäms genom en parallell tillförsel av ^{13}C-fenylalanin till den enterala nutritionen.

Helkropps proteinkinetiken beräknas genom bestämning av ”rate of appearance” R_a för fenylalanin (=proteindegradation) och oxidering av fenylalanin till tyrosin varefter ”rate of disappearence” Rd för fenylalanin (=proteinsyntes) kalkyleras. För denna bestämning används deuterium-märkt fenylalanin och tyrosin (5d-fenylalanin, 2d-tyrosin och d4-tyrosin).

Vid beräkningarna av splanknikus extraktion subtraheras det fenylalanin som tillförts med nutritionen och som når provtagnings-poolen.

Protokoll

Patienter som erhåller full nutrition kan ingå i delstudie A. Om en patient erhåller dialysbehandling skall det noteras och diaslysflöden inklusive dragning skall inte ändras under de 4 timmar provtagning sker varje nutritionsdygn och prov från dialysvätskan tas. Patienterna studieras under 2 på varandra följande dygn då de i randomiserad ordning erhåller 50% respektive 100% av EE vid studiens början. Randomisering sker blindat genom dragning av slutna kuvert i grupper om 4. Om möjligt utförs indirekt kalorimetri omedelbart före studien och även varje dag under studien.

Tillförseln av tracer påbörjas 5 timmar före mättillfället för helkropps proteinomsättning. Tiden är vald för att optimera möjligheten till att upptaget är konstant, utan att risken för re-cirkulation av isotopmärkt aminosyra blir onödigt stor. Blodprovstagning sker före start av tillförsel av det ^{13}C -märkta fenylalaninet samt var 10:e minut under den sista halvtimmen av nutritionsdygnet.

Deuterium-märkt fenylalanin infunderas tillsammans med deuterium-märkt tyrosin, vilket är oxidationsprodukten av fenylalanin. Dessa aminosyror tillförs som primade konstanta infusioner under totalt 180 minuter. Blodprovstagning sker före infusionen samt vid 4 tillfällen under infusionens sista halvtimme.

Vid sidan av mättillfällena innehåller studien inget protokoll. Vid varje provtagningstillfälle insamlas patientdata omfattande patientkaraktäristika, diagnos, pågående nutrition, medicinering, rutinlaboratorieprover, pågående dialys etc genom ett utdrag (genom utskrift) ur den elektroniska patientjournalen.

I delstudie B erhåller friska försökspersoner kontinuerlig näringstillförsel i nivå med 100 % av sin basala energifrisättnig genom en tunn nasogastrisk sond under 24 timmar. Efter 12 timmar adderas ^{13}C -märkt fenylalanin som tillsats till den enterala nutritionen. Därefter sker också blodprovstagning var 30:e minut under 12 timmar.

Inklusionskriterier

Kritiskt sjuka patienter som erhåller full nutrition kan ingå i studie A och friska försökspersoner i delstudie B.

Exklusionskriterier

Barn (<18år), frånvaro av medgivande, ej full enteral nutrition, behov av blodtransfusion under studieperioden.

Studiestorlek, statistik

Någon skillnad mellan att erhålla 50% av nutritionen före eller efter 100% av nutritionen sågs ej i den tidigare studien, därför förväntas inte heller någon sådan skillnad i denna studie. Patienterna blir sina egna kontroller. Därför bedöms att resultaten kan bli konklusiva om minst 6 evaluerbara patienter finns i vardera gruppen. Totalt kommer det att finnas 12 evaluerbara patienter, som kommer att jämföras med sig själva. Om de kan betraktas som en grupp kommer en skillnad (eller avsaknaden av skillnad) av uppmätta variabler om 75% av standarddeviationen att kunna säkerställas med 80 % statistisk power.

Etiska överväganden

Patienterna kommer inte att utsättas för någon risk. Blodprovstagningen kommer att totalt vara < 50 mL. Administreringen av aminosyror märkta med stabila isotoper sker i liten mängd och är helt ofarlig. Patienterna utsätts inte för några stick eller andra obehag. Näringstillförseln kommer att halveras under 1 dygn, vilket är en möjlig fördel eller nackdel, vilket är studiens kliniska frågeställning. Deltagandet i studien innebär att nutritionsbehandlingen totalt sett sker med stor noggrannhet och under speciell övervakning. Totalt sett innebär studien att viktig kunskap kan erhållas utan risk för patienterna och utan någon säker nackdel.

Referenser

1. Rooyackers O, Kouchek-Zadeh R, Tjader I, Norberg A, Klaude M, Wernerman J: **Whole body protein turnover in critically ill patients with multiple organ failure**. *Clin Nutr* 2015, **34**(1):95-100.
2. Liebau F, Wernerman J, van Loon LJ, Rooyackers O: **Effect of initiating enteral protein feeding on whole-body protein turnover in critically ill patients**. *Am J Clin Nutr* 2015, **101**(3):549-557.
3. Liebau F, Sundstrom M, van Loon LJ, Wernerman J, Rooyackers O: **Short-term amino acid infusion improves protein balance in critically ill patients**. *Crit Care* 2015, **19**:106.
4. Berg A, Rooyackers O, Bellander BM, Wernerman J: **Whole body protein kinetics during hypocaloric and normocaloric feeding in critically ill patients**. *Crit Care* 2013, **17**(4):R158.
5. Arabi YM, Tamim HM, Dhar GS, Al-Dawood A, Al-Sultan M, Sakkijha MH, Kahoul SH, Brits R: **Permissive underfeeding and intensive insulin therapy in critically ill patients: a randomized controlled trial**. *Am J Clin Nutr* 2011, **93**(3):569-577.
6. Krishnan JA, Parce PB, Martinez A, Diette GB, Brower RG: **Caloric intake in medical ICU patients: consistency of care with guidelines and relationship to clinical outcomes**. *Chest* 2003, **124**(1):297-305.
7. Marik PE, Pinsky M: **Death by parenteral nutrition**. *Intensive Care Med* 2003, **29**(6):867-869.
