# Supplementary material for: Whole-body protein kinetics in critically ill patients during 50 or 100% energy provision by enteral nutrition: A randomized cross-over study
Source: PLoS One. 2020 Oct 5;15(10):e0240045. doi: 10.1371/journal.pone.0240045 (PMC7535026; doi:10.1371/journal.pone.0240045)
Supplement: S2 File — (DOCX) [file pone.0240045.s003.docx]

**Whole body protein kinetics in critically ill patients during 50 or 100% energy provision by enteral nutrition: A pilot randomized cross-over study.**

English language translation of the application to the regional ethical review board in Stockholm County 2016-01-03 Dnr 2016/76-31. Translation of ethical application sections 1-7 page 5-16; study protocol submitted as supplementary material page 30-33 of original document in pdf format. Information concerning Study B (a separate study submitted on the same application) has been omitted from parts of the translation.

1. Information about the sponsor

1:1 Study sponsor

Name: Stockholm County Council

Adress: Department of Anaesthesia, Karolinska University Hospital Huddinge, 14186 Stockholm

1:2 Qualified representative of the study sponsor

Name: Patrik Rossi

Title: Head of department

Adress: Department of Anaesthesia, Karolinska University Hospital Huddinge, 14186 Stockholm

1:3 Investigator with chief responsibility for project conduct (contact person)

Name: Jan Wernerman

Title: Professor, senior consultant

Adress: Department of Anaesthesia, Karolinska University Hospital Huddinge, 14186 Stockholm

E-mail: [jan.wernerman@karolinska.se](mailto:jan.wernerman@karolinska.se)

Phone: 08-58586395

Mobile: 0707461000

1:4 Other participants (listed in supplementary material)

1:5 Declaration of access to required resources during project conduct

The necessary resources are available within ALF grants and FoUU grants to the researcher. See supplementary certificate of resources.

1:6 Application to other authorities under certain conditions

2. Information about the project (Lay summary)

2:1 Summary of the research project

Several authors claim that hypocaloric nutrition is advantageous compared to full nutrition in critically ill patients (Arabi et al, Am J Clin Nutr 2011;93:569-577, Krishnan et al, Chest 2003;124:297-305, Marik & Pinsky, ICM 2003;29:867-869). This has resulted in different recommendations for nutritional therapy to critically ill patients in Europe and North America. In an earlier study, we did not find any support for this hypothesis in neurosurgical patients with parenteral nutrition (Berg et al, Crit Care 2013;17:R158).

In that study we compared full and half nutrition to neurosurgical patients who received parenteral nutrition in this way. The nutrition was composed of a standard product and dosed according to indirect calorimetry. Patients received 50 or 100% of measured energy expenditure during 24 hours in randomized order. Measurements of whole body protein kinetics were conducted during the last two hours of the respective nutrition regime. Results demonstrated an improved whole body protein balance at 100%, which indicates an advantage of full nutrition. Due to it’s limited size the study could not assess clinical outcomes or complications.

We are now planning to conduct the same study protocol but with enteral nutrition in a mixed group of critically ill patients. The technique to assess nutritional therapy with whole body protein turnover has previously not been used in critically ill patients with enteral nutrition. Therefore we have recently conducted several validations of this technique with enteral nutrition (EPN 2011/2029, EPN 2014/116). The results are partially published and demonstrate that whole body protein turnover is relatively constant during the acute phase of critical illness, the first week of intensive care unit stay (Liebau et al, Crit Care 2015:19:106)

We now wish to investigate the effect of a standard formula of enteral nutrition in different amounts on whole body protein balance (Study A), and in healthy subjects perform a complementary study on the uptake of isotopically labeled amino acids from the gut (Study B, complement to EPN 2014/116).

2:2 What is the scientific hypothesis behind the project design?

Study A. What is the effect of hypocaloric and normocaloric enteral nutrition of standard type on whole body protein balance in critically ill patients?

Study B. How does the uptake of an isotopically labeled amino acid correspond with the uptake of protein content from a standard product for enteral nutrition in healthy individuals. Is the variability equally large in healthy subjects compared to ICU patients?

2:3 Describe any results from relevant animal studies

Not relevant to the project in question.

2:4 Provide a general description of the investigation procedure, data collection and the type of data collected.

Study A. Patients receiving full nutrition can participate in the study. If a patient has continuous renal replacement therapy this is documented and dialysis flow and ultrafiltration are to be kept constant during the 4 hour sampling which is conducted each 24 hour nutritional period and a sample of dialysis effluent is taken. Patients are studied on 2 following days where they in randomized order receive 50% or 100% respectively of measured energy expenditure at the start of the study. Randomization is blinded by drawing of sealed opaque envelopes in groups of 4. If possible indirect calorimetry is performed immediately prior to the study and every following study day.

The supplementation of 13C-labeled phenylalanine is started 8 hours prior to the measurement of whole body protein turnover. The timing is chosen to optimize the conditions for constant uptake while reducing the risk of excessive recirculation of isotopically labeled amino acids. Blood sampling is conducted before starting the infusion of 13C-labeled phenylalanine and every 10 minutes during the last half hour of the nutrition period.

Deuterium-labeled phenylalanine is infused together with deuterium-labeled tyrosine, which is the oxidation product of phenylalanine. These amino acids are given as primed constant infusions during 180 minutes. Blood sampling is performed prior to the infusions and at 4 time points during the last half hour of the infusion.

Whole body protein kinetics are determined as follows. Infusions of phenylalanine and tyrosine are started (bolus: 13C-phenylalanine/2H5-phenylalanine 0.5 mg/kg, 2H4-tyrosine 0.15 mg/kg, 2H2-tyrosine 0.3 mg/kg followed by a continuous intravenous infusion during approximately five hours of 2H5-phenylalanine 0.5 mg/kg/h, 2H2-tyrosine 0.3 mg/kg/h and a continuous enteral infusion of 13C-phenylalanine 0.5 mg/kg/h. At steady state after 2 hours the measurements are performed during the following 30 minutes. Blood samples of 4 mL are drawn at a total of 10 occasions. For the patients this does not require any additional venipuncture as a central venous catheter, arterial line and nasogastric tube are already in situ.

The total volume of blood sampled in study A is 80 mL.

2:5 Describe if biological materials will be stored in a biobank

Collected material is stored in a biobank at Karolinska University Hospital Huddinge until the study is published, when remaining samples are disposed. Samples are de-identified and only labeled by numbers. The list connecting patient data with sample numbers are only available at a location of the study sponsor.

2:6 Documentation, data protection and archiving

Collected patient and subject data and the analysis results are stored with the investigator and do not leave specific locked areas of the hospital prior to de-identification. The material will be completely de-identified when the data compilation and publication is complete.

2:7 Describe prior experience (your own or others) of the applied procedure, technique or therapy

The investigation is a continuation of studies Dnr 290/03, 2006-1499, 2009-1647, 2011-2029, 2014-116. The primary investigator has extensive experience of metabolic studies in critically ill patients and healthy subjects.

3. Information about research subjects

3:1 How are the research subjects recruited?

Patients are recruited as research subjects in the intensive care unit if they are circulatory stable and receiving full enteral nutrition, healthy subjects are recruited among individuals who spontaneously show interest in participating in metabolic studies.

3:2 Provide the relation between the primary investigator and research subjects

Physician.

3:3 Describe the statistical considerations for the population sample size

Study A. No difference was observed in the response to receiving 50% of nutrition before or after 100% of nutrition in the previous study [Berg et al, Crit Care 2013;17(4):R158], therefore no difference is expected in this study. Patients serve as their own controls. From these observations it is determined that the results may be conclusive with 6 evaluable patients in each group. In total there will be 12 patients with their own prior results as control. If these can be considered a group a difference (or absence of difference) of measured variables corresponding to 75% of the standard deviation can be detected with 80% power.

3:4 Can the research subjects be included in other studies in parallell to or in conjunction with this study? If so, what type of research?

There are no barriers for these patients to participate in other studies. Subjects should not have participated in other studies 1 month before this study.

3:5 What type of insurance is provided for research subjects participating in this project?

Patients and research subjects are covered by patient insurance.

3:6 What type of economic reimbursement or any other type of advantage is provided to the research subjects of this project?

No economic reimbursement is provided.

4. Information and consent

4:1 Procedure for and content of the information provided when research subjects are asked to participate

The patient or closest relative is informed orally and in writing about participation in Study A. See supplementary patient information.

4:2 How and from whom is consent provided?

See 4:1

5. Ethical considerations

5:1 Describe all potential risks of participation

The intravenous provision of isotopically labeled amino acids is not associated with any risks.

Patients in Study A have routine venous- and arterial catheters, therefore there is no risk associated with additional vascular access.

In addition, the patients in Study A will receive 50% of prescribed nutrition for 24 hours, which deviates from the standard routine of Karolinska Huddinge, but is standard practice at several institutions around the world, for example in North America.

In Study A the total volume of blood sampling will be less than 80 mL, which is only associated with minimal risk.

5:2 Describe any potential advantages that participation in the research project may entail (especially in therapeutic interventions)

The research subjects have no potential personal gain from participation in the study.

5:3 Identify and detail any ethical problems (advantages/disadvantage) in a broader perspective that may result from the project

The benefit of the project, i.e. the knowledge acquired is large for the group of critically ill patients. Today there is a lack of adequate methodology to evaluate how nutrition should be dosed in acutely ill intensive care unit patients. The extension and completion of the study in question entails valuable steps to acquiring this knowledge. Therefore the advantages from a general perspective are large in proportion to the risks that research subjects and patients are exposed to and the violation of their integrity.

6. Reporting of results

6:1 How is the study sponsor and investigators guaranteed access to data (this is reported in contract research) and who is responsible for data analysis and reporting of results?

Not applicable.

6:2 How will the results be made public? Will the study be submitted for publication in an academic journal or published in another way?

The results are intended to be published in an academic journal.

6:3 In which way is the research subjects right to integrity guaranteed when the material is made public/published?

The patients and research subjects will not be able to be identified in the publicized material.

7. Reporting of any economic relations and financial disclosures

7:1 Reporting for contract research

7:2 Describe any financial contracts with the sponsor or other financial sources (name, amount)

Not applicable

7:3 Describe the sponsors, chief investigators and participating researchers personal interests (Declaration of stock holdings, employment, consulting positions in financing companies, personal company that may (directly or indirectly) have economic gain from this research.

Not applicable.

Research plan

Whole body protein turnover in critically ill patients during normocaloric and hypocaloric enteral nutrition

Chief Investigator: Jan Wernerman

**Background**

Protein turnover and the effect of nutrition have traditionally been determined using nitrogen balance techniques. These techniques have several limitation, particularly when used in circumstances where the individuals investigated are not adapted to the caloric or energy intake which is to be measured. Nitrogen balance yields reliable results when evaluated for a isocaloric and isonitrogenic diet that the individual investigated is fully adapted to.

Those necessary conditions however do not exist in illness. Nonetheless, current recommendations for protein delivery in illness are largely based on nitrogen balance studies.

A different approach is to measure protein content in the body. Available methods are, inter alia, MRT, ultrasound, and CT scanning. Technical advances of these techniques will probably allow measurements of small differences in the future. One difficulty which will probably be overcome in the future is the variable water content of body tissues.

The most promising technique today is to measure whole-body protein kinetics using an isotope labeled amino acid. This technique allows to differentiate between protein synthesis, degradation, and substrate oxidation, which is not possible with any other technique. Furthermore, it allows to measure these quantities even on the level of individual tissues, using blood vessel cannulation and/or biopsies. In its simplest form, the technique consists of injection of an isotope labeled amino acid and sequential sampling after an equilibration period. Depending on the choice of isotope labeling, collecting expiratory CO_2_ may be necessary to determine substrate oxidation. The most relevant limitation in the simple form of this technique is that only those tissues whose amino acid turnover equilibrates with the sampling pool can be investigated.

In the postabsorptive state, this information is sufficient. Similarly, when an individual receives parenteral nutrition by constant intravenous infusion during the measurement period [[1](#_ENREF_1)]. The situation is more complicated when an individual is fed enteral nutrition. Even if the rate of intake of enteral nutrition is kept constant, this does not mean that uptake into the sampling pool (usually blood plasma) remains unchanged. We have shown that after 6 hrs of constant supply of isotope labeled milk protein, there is a constant uptake of isotope labeled amino acid into the sampling pool [[2](#_ENREF_2)]. This experiment was done with supply of a relatively small fraction of total nutritional requirement. Such a constant uptake could be shown in both healthy subjects and in ICU patients. However, splanchnic extraction fraction was high, and was disparate in healthy subjects and ICU patients

Furthermore, we have shown that, after an equilibration period, the uptake into the sampling pool of a free isotope labeled amino acid is identical to that of labeled amino acid from intrinsically labeled milk protein during concomitant enteral infusion (Fig.2) [[3](#_ENREF_3)]. This means that for further investigations we are not bound to use special proteins intrinsically labeled with isotopes, but can use standard nutrition with a parallel infusion of free isotope labeled amino acid.

In the pilot studies referred above, a relatively low supply of enteral nutrition was used and a relatively high splanchnic extraction was seen. It is not clear whether this is similarly true for full nutrition. A validation of this was done in a pilot study (EPN 2014/116). The results show a relevant variation over time in ICU patients. It is unknown whether this is physiological, i.e. found in healthy individuals under similar conditions, or specific to critically ill individuals.

In another previous study, we have compared full-dose vs. half-dose parenteral nutrition in neurosurgical patients [[4](#_ENREF_4)]. Nutrition was given as a standard product and its dose was adapted to energy expenditure (EE) measured by indirect calorimetry. Patients were randomized to receive either first 50% of EE for 24 hrs and then 100% of EE, or the reverse. Measurements of protein turnover was performed during the last 2 hrs of each nutritional regimen. We found that protein balance is more favorable with a supply of 100% of EE, while substrate oxidation is not different between regimens. This shows that, with a protein supply of 1.1 g/kg/day and energy supply of 100 of measured EE, there is no oxidation of surplus protein supplied with parenteral nutrition. The motivation for that study was that a number of authors claim that hypocaloric nutrition is advantageous compared with full nutrition in critically ill patients [[5-7](#_ENREF_5)]. In our study we did not find support for that hypothesis in neurosurgical patients on parenteral nutrition. The result showed a better protein balance with supply of 100%, which supports supplying full-dose nutrition. However, no outcome measures or complications were evaluated, as that would have required a very different dimension of the investigation.

We are now planning to run an identical protocol as in the neurosurgical patients, but using a supply of enteral nutrition. The study questions are identical to the previous experiments, with the additional question of measuring splanchnic extraction fraction of dietary amino acid.

**Methods**

Uptake and utilization of enteral nutrition is determined by parallel supplementation of 13C-phenylalanine to standard enteral nutrition.

Whole body protein kinetics are calculated by determination of “rate of appearance” Ra for phenylalanine (= protein degradation) and oxidation of phenylalanine to tyrosine from which the “rate of disappearance” Rd for phenylalanine (= protein synthesis) is calculated. For this purpose deuterium-labeled phenylalanine and tyrosine (5D-phenylalanine, 2D-tyrosine and 4D-tyrosine).

For the calculation of splanchnic extraction the dose of phenylalanine reaching the central compartment is subtracted from the dose provided with the nutrition.

**Protocol**

Patients who are fully fed by the enteral route can be included in Study A. If a patient is treated with renal replacement therapy this is noted in the case report form, and dialysate flow rates including ultrafiltration are to be kept constant during the four hour sampling period when samples from the dialysis effluent are taken. Patients are studied during two consecutive days and receive, in randomized order, either 50% or 100% of measured EE at the start of the study for 24 hours. Randomization is performed by drawing sealed envelopes in groups of four. If possible, indirect calorimetry is performed immediately before the start of the experiment and then each day during the study period.

The tracer supplementation is started 5 hours before measurement of whole-body protein turnover. The timing is chosen to optimize the chance that there is a constant uptake without unnecessarily raising the risk of tracer recirculation. Blood samples are drawn before the start of ^13^C-Phe and every 10 minutes during the last half hour of each 24 hour nutrition period. Deuterium labeled phenylalanine is infused together with deuterium labeled tyrosine, which is the oxidation product of phenylalanine. These infusions are given as primed constant intravenous infusions for a total of 180 minutes. Blood sampling is done before starting infusions and at four time points during the last half hour of infusion.

Apart from the feeding periods and tracer investigations there is no further experimental protocol. Data collected comprise patient characteristics, diagnoses, ongoing nutrition, medications, clinical routine lab tests, ongoing dialysis etc extracted from the electronic medical records.

**Inclusion criteria**

Critically ill patients receiving full enteral nutrition are eligible for Study A.

**Exclusion criteria**

Pediatric subjects (<18 years of age), lack of informed consent, not established full enteral nutrition, blood transfusions during study measurements.

**Sample size, statistics**

No difference was observed in the response to receiving 50% of nutrition before or after 100% of nutrition in the previous study [Berg et al, Crit Care 2013;17(4):R158], therefore no difference is expected in this study. Patients serve as their own controls. From these observations it is determined that the results may be conclusive with 6 evaluable patients in each group. In total there will be 12 patients with their own prior results as control. If these can be considered a group a difference (or absence of difference) of measured variables corresponding to 75% of the standard deviation can be detected with 80% power.

**Ethical considerations**

Patients will not be exposed to any risk. The total volume of blood samples will be <50 mL. Amino acids labeled with stable isotopes are administered in small amounts and is not associated with any potential harm. Patients will not be exposed to any painful procedures. The delivery of nutrition will be halved for one day, a possible advantage or disadvantage, which is the hypothesis investigated by the study. Participation in the study will entail that the nutritional therapy is conducted with a high degree of precision and monitoring. In summary, the study has the potential to elucidate important knowledge without risk or any certain disadvantage to the patients.
