# Supplementary material for: Whole-body protein kinetics in critically ill patients during 50 or 100% energy provision by enteral nutrition: A randomized cross-over study
Source: PLoS One. 2020 Oct 5;15(10):e0240045. doi: 10.1371/journal.pone.0240045 (PMC7535026; doi:10.1371/journal.pone.0240045)
Supplement: Attachment — Submitted filename: PLOS ONE Response to reviewers V2.docx [file pone.0240045.s005.docx]

**Response to reviewers**

Reviewer #3: The authors have made a commendable effort to respond to previous reviewers' comments. Although I agree that this is an important and novel study, I am still not satisfied with the responses to my previous comments relating to whether this is a pilot study or a small trial.

"Some recent reviews ... highlighted that sometimes a small underpowered effectiveness study is labelled as a pilot or feasibility study. There is therefore a need to raise awareness of the difference between a pilot study which is designed to clarify areas of uncertainty, and a small underpowered study labelled as a pilot which does not comply with definitions and is not reported according to the CONSORT guidance" - <https://pilotandfeasibilitystudies.qmul.ac.uk/introduction/>

The study still includes descriptions which present it as if it were a pilot study, e.g. the title, abstract, discussion, without reporting the requisite feasibility outcomes. I would recommend that the authors review the explanations and references provided in the link above to help with clarifying these issues.

One additional minor comment is that Table 4 could be improved by adding a column withe the mean difference and 95% confidence intervals for the differences. Additionally, the 50%EN and 100%EN columns should show the means and standard errors rather than the means and ranges - this is the standard format for reporting tables of main results for statistical inference.

*The authors would again like to thank Reviewer #3 for insightful comments. The study is correctly identified as a small clinical trial, the word “pilot” (inappropriately) used to indicate the exploratory nature of the trial design and focus on physiological endpoints. The manuscript has been revised to avoid any ambiguity in this regard. We are aware of the limitations of a small and heterogenous sample, and hope that the reviewer feels that this has been adequately addressed in the discussions section and previous replies to the reviewers.*

*Table 4 has also been revised according to recommendations.*
